# Supplementary material for: Interferon induced circRNAs escape herpesvirus host shutoff and suppress lytic infection
Source: bioRxiv. 2023 Sep 7:2023.09.07.556698. Preprint. [Version 1] doi: 10.1101/2023.09.07.556698 (PMC10602050; doi:10.1101/2023.09.07.556698)
Supplement: Supplement 1 [file media-1.pdf]

Supporting Information for

Interferon induced circRNAs escape herpesvirus host shutoff and suppress lytic infection.

Sarah E. Dremel[1,2], Takanobu Tagawa[1,2], Vishal N. Koparde[3,4], Jesse H. Arbuckle[5], Thomas M. Kristie[5], Laurie T. Krug[2], Joseph M. Ziegelbauer[2]*

1. These authors contributed equally

2. HIV and AIDS Malignancy Branch, National Cancer Institute, Bethesda, MD, United States

3. CCR Collaborative Bioinformatics Resource, Center for Cancer Research, National Cancer Institute, National Institutes of Health, Bethesda, MD, United States

4. Advanced Biomedical Computational Sciences, Frederick National Laboratory for Cancer Research, Leidos Biomedical Research, Inc., Frederick, MD, United States

5. Laboratory of Viral Diseases, National Institute of Allergy and Infectious Diseases, Bethesda, MD, United States

*Corresponding Author: Joseph M. Ziegelbauer (ziegelbauerjm@nih.gov)

This PDF file includes:

Supplementary methods
Legend for Table S1
Tables S2 to S3
Legends for Datasets S1 to S6
Figures S1-1, S1-2, S1-3, S1-4, S1-5, S 3-1, S 4-1, S4-2, S4-3
SI References

Other supporting materials for this manuscript include the following:

Table S1
Datasets S1 to S6

SUPPLEMENTARY METHODS

Immune stimulation of cell culture models
Confluent monolayers of MRC-5 and LEC were treated with a variety of immunostimulants. 10 ug/mL lipopolysaccharide (Millipore Sigma L3024), 4 ug/mL CpG DNA (IDT), 10 ug/mL poly I:C (Millipore Sigma #P1530), or 25 ng/mL recombinant human IFN-β and γ (Peprotech #300-02BC and #300-02) was added to culture media. After the indicated treatment time (8, 24, 48, or 72 hours), RNA was isolated from the cell fraction. For Akata-, Daudi, or BJAB, 10 ug/mL lipopolysaccharide, 10 ug/mL polyI:C, 10 ng/mL IFN-β, or 500 ng/mL IFN-γ were added to culture media. After 24 hours RNA was isolated from the cell fraction.

Immune stimulation of PBMCs
Human peripheral blood mononuclear cells (PBMCs) were prepared from buffy coats. Buffy coats from two different donors were purified with Ficoll-Paque PLUS (GE Healthcare #17144003). PBMCs were treated with red blood cell lysis buffer (BioLegend #420301) and washed three times with phosphate-buffered saline (Gibco #10010023). 10 or 30 ng/mL recombinant human IFN-β (Peprotech #300-02BC) was added to the culture media. After 24 hours RNA was isolated from the cell fraction.

***In silico* circRNA-miRNA-mRNA network predictions**
Four commonly-regulated human circRNAs (hsa_circ_0001730, hsa_circ_0006990, hsa_circ_0006646, hsa_circ_0001769) were examined for miRNA-binding sites using CircInteractome (1). Experimentally supported miRNA-mRNA interactions were then added to the network based on DIANA-TarBase v8.0 (2). Cytoscape 3.10 was used for visualizing the network (3). Overrepresentation analysis of predicted target miRNAs was performed with miEAA 2.0 (4). Enriched Gene Ontology terms (http://geneontology.org), p-values, and miRNAs that were predicted to be regulated by circRNAs are shown.

Overrepresentation analysis (ORA)
ORA was performed using a list of genes colinear to circRNAs detected in a given model (>10 raw BSJ read counts). ORA was performed using WebGestalt (5) and plotted using SRplot (https://www.bioinformatics.com.cn/en).

Ingenuity pathway analysis (IPA)
IPA (Qiagen) was performed on *de novo* lytic infection RNA-Seq datasets for HSV-1 (MRC-5 infected at MOI (multiplicity of infection) of 10 for 12 hours, 4 biological replicates), HCMV (MRC-5 infected at MOI of 3 for 72 hours, 2 biological replicates), and KSHV (LEC infected at MOI of 1 for 72 hours, 2 biological replicates). Log_2 fold change (Log_2FC, Infected/Uninfected) was calculated from ERCC normalized data. Only genes with an average Log_2FC >1 were used for IPA. Bubble plots were generated using SRplot (https://www.bioinformatics.com.cn/en).

SUPPORTING DATASETS

Supporting Dataset 1. Transcriptomic data from HSV-1 human infection models
Transcriptomic data from bulk RNA-Seq data for MRC-5 infected with HSV-1 strain KOS at MOI of 10 plaque-forming unit (PFU)/cell for 12 or 24 hours. Data was mapped to a concatenated genome assembly containing hg38 (gencode.v36), KT899744.1, and ERCC (External RNA Controls Consortium) spike-ins. Tab 1-GenePlotter) Query ERCC normalized gene expression data from the samples by inputting gene symbol. Tab 2-CircPlotter) Query ERCC normalized back splice junction (BSJ) data from the samples by inputting circBase annotation. Tab 3-Summary data) RNA Star 2-pass mapping log output file information, including input reads and those mapped to the genome assemblies. Tab 4-Raw counts) CircRNA counts are generated by CircExplorer3 (6) mapping and include only BSJ reads. All other gene counts are outputs from RNA STAR GeneCount (per gene read counts). Tab 5-ERCC norm counts) BSJ or gene counts normalized using ERCC spike-in reads. Tab 6-Log2FC) Log_2FC (Infected/Uninfected) for ERCC normalized counts.

Supporting Dataset 2. Transcriptomic data from HCMV infection models
Transcriptomic data from bulk RNA-Seq data for MRC-5 infected with HCMV strain TB40/E at MOI of 3 PFU/cell for 24 or 72 hours (7). Data was mapped to a concatenated genome assembly containing hg38 (gencode.v36) and NC_006273.2. Tab 1-GenePlotter) Query TPM (transcript per million) normalized gene expression data from the samples by inputting gene symbol. Tab 2-CircPlotter) Query TPM normalized BSJ data from the samples by inputting circBase annotation. Tab 3-Summary data) RNA Star 2-pass mapping log output file information, including input reads and those mapped to the genome assemblies. Tab 4-Raw counts) CircRNA counts are generated by CLEAR (CircExplorer3) mapping and include only BSJ reads. All other gene counts are outputs from RNA STAR GeneCount (per gene read counts). Tab 5-TPM norm counts) BSJ or gene counts normalized as TPM. Tab 6-Log2FC) Log_2FC (Infected/Uninfected) for TPM normalized counts.

Supporting Dataset 3. Transcriptomic data from KSHV infection models
Transcriptomic data from bulk RNA-Seq data for i. LEC infected with KSHV strain BAC16 at MOI of 1 PFU/cell for 24 or 72 hours or ii. iSLK-BAC16 reactivated with 1 mM Sodium Butyrate 1 ug/mL Doxycycline for 24 or 72 hours. Data was mapped to a concatenated genome assembly containing hg38 (gencode.v36), NC_009333.1, and ERCC spike-ins. Tab 1-GenePlotter) Query ERCC normalized gene expression data from the samples by inputting gene symbol. Tab 2-CircPlotter) Query ERCC normalized BSJ data from the samples by inputting circBase annotation. Tab 3-Summary data) RNA Star 2-pass mapping log output file information, including input reads and those mapped to the genome assemblies. Tab 4-Raw counts) CircRNA counts are generated by CLEAR (CircExplorer3) mapping and include only BSJ reads. All other gene counts are outputs from RNA STAR GeneCount (per gene read counts). Tab 5-ERCC norm counts) BSJ or gene counts normalized using ERCC spike-in reads. Tab 6-Log2FC) Log_2FC (Infected/Uninfected) for ERCC normalized counts.

Supporting Dataset 4. Transcriptomic data from HSV-1 mouse infection models
Transcriptomic data from bulk RNA-Seq data for i. mice trigeminal ganglia (TG) latently infected with HSV-1 strain 17 for 4 weeks, or ii. TG explants from mice infected with HSV-1 strain 17 for 5 weeks and grown in the presence or absence of 2 uM JQ1 (Selleckchem, Houston TX) for 12 hours. Data was mapped to a concatenated genome assembly containing mm39 (gencode.vM29), NC_001806.2, and ERCC spike-ins. Tab 1-GenePlotter) Query ERCC normalized gene expression data from the samples by inputting gene symbol. Tab 2-CircPlotter) Query ERCC normalized BSJ data from the samples by inputting circBase annotation. Tab 3-Summary data) RNA Star 2-pass mapping log output file information, including input reads and those mapped to the genome assemblies. Tab 4-Raw counts) CircRNA counts are generated by CLEAR (CircExplorer3) mapping and include only BSJ reads. All other gene counts are outputs from RNA STAR GeneCount (per gene read counts). Tab 5-

ERCC norm counts) BSJ or gene counts normalized using ERCC spike-in reads. Tab 6-Log2FC) Log_2FC (Infected/Uninfected) for ERCC normalized counts.

Supporting Dataset 5. Transcriptomic data from MHV68 infection models
Transcriptomic data from bulk RNA-Seq data for (i) NIH 3T3 infected with MHV68 strain H2B-YFP MOI of 5 PFU/cell for 6 or 18 hours, or (ii) A20 HE-RIT reactivated with 5 ug/ml Doxycycline and 20 ng/ml 12-O-tetradecanoylphorbol-13-acetate (TPA) for 6 or 24 hours. Data was mapped to a concatenated genome assembly containing mm39 (gencode.vM29), MH636806.1, and ERCC spike-ins. Tab 1-GenePlotter) Query ERCC normalized gene expression data from the samples by inputting gene symbol. Tab 2-CircPlotter) Query ERCC normalized BSJ data from the samples by inputting circBase annotation. Tab 3-Summary data) RNA Star 2-pass mapping log output file information, including input reads and those mapped to the genome assemblies. Tab 4-Raw counts) CircRNA counts are generated by CLEAR (CircExplorer3) mapping and include only BSJ reads. All other gene counts are outputs from RNA STAR GeneCount (per gene read counts). Tab 5-ERCC norm counts) BSJ or gene counts normalized using ERCC spike-in reads. Tab 6-Log2FC) Log_2FC (Infected/Uninfected) for ERCC normalized counts.

Supporting Dataset 6. Transcriptomic data from interferon stimulation experiments
Transcriptomic data from bulk RNA-Seq data for MRC-5, LEC, or Akata- treated with IFN-β and -γ for 48 hours. Data was mapped to a concatenated genome assembly containing hg38 (gencode.v36) and ERCC spike-ins. Tab 1-GenePlotter) Query ERCC normalized gene expression data from the samples by inputting gene symbol. Tab 2-CircPlotter) Query ERCC normalized BSJ data from the samples by inputting circBase annotation. Tab 3-Summary data) RNA Star 2-pass mapping log output file information, including input reads and those mapped to the genome assemblies. Tab 4-Raw counts) CircRNA counts are generated by CLEAR (CircExplorer3) mapping and include only BSJ reads. All other gene counts are outputs from RNA STAR GeneCount (per gene read counts). Tab 5-ERCC norm counts) BSJ or gene counts normalized using ERCC spike-in reads. Tab 6-Log2FC) Log_2FC (Infected/Uninfected) for ERCC normalized counts.

SUPPLEMENTARY TABLES

Supplementary Table 1. Differentially expressed circRNAs

Summary table of all circRNAs which met our significance threshold to be called as differentially expressed. CircRNAs are annotated relative to their colinear gene and back splice junction and cross-referenced via circBase (human) or circAtlas (mouse).

Supplementary Table 2. Oligos used in this study

Sequences of oligos used for qPCR or other applications in this study.

Target	Target Species	Forward Primer	Reverse Primer	Notes
**US1 (ICP22)**	HSV-1	TTTGGGGAGTTTGACTGGAC	CAGACACTTGCGGTCTTCTG	
**UL23 (tk)**	HSV-1	ACCCGCTTAACAGCGTCAACA	CCAAAGAGGTGCGGGAGTTT	Used to detect HSV-1 genome copies
**UL44 (gC)**	HSV-1	GTGACGTTTGCCTGGTTCCTGG	GCACGACTCCTGGGCCGTAACG	
**circMAN1A2**	Human	TAGGACATATGGGTGGGGAC	TGCTTCTTCCAAGGCCTTCT	Divergent Primers (hsa_circ_0000116)
**circEPSTI1**	Human	AAGCTGAAGAAGCTGAACTC	GTGTATGCACTTGTGTATTGC	Divergent Primers (hsa_circ_0000479)
**circRELL1**	Human	ATGTCTGTTAGTGGGGCTGA	TATCTGCTACCATCGCCTTT	Divergent Primers (hsa_circ_0001400)
**circTNPO3**	Human	GCTAATCGGCGCACAGAAAT	GGTCTGAGATCTCCCATGCA	Divergent Primers (hsa_circ_0001741)
**circECE1**	Human	ACCTCTGGGAACACAACCAA	GCCGTTGGGGTATGCGTC	Divergent Primers (hsa_circ_0002402)
**circIPO7**	Human	GGGCCAGATGAAGAAGGTAGT	GAGCCTGCATTACAGGTCTGAT	Divergent Primers (hsa_circ_0005092)
RELL1	Human	GCAGTGGCACAGAGTAGCAG	CAGTGCAGCCTTACCAGTTG	Primers span exon-exon junction
EPSTI1	Human	GACAGAAGTGCCTGTCAAAGTG	GCCGTTTCAGTTCCAGTAATTC	Primers span exon-exon junction
ISG15	Human	GAGAGGCAGCGAACTCATCT	CTTCAGCTCTGACACCGACA	
**18S rRNA**	Human	GTAACCCGTTGAACCCCATT	CCATCCAATCGGTAGTAGCG	
GAPDH	Human	AGCCTCAAGATCATCAGCAATG	ATGGACTGTGGTCATGAGTCCTT	Primers span exon-exon junction
***SHFL* (Shiftless)**	Human	GGCTCTGATGAGGAAATTCGGC	TCCTGGGCATCCAGATCGTTAC	
**U6 snRNA**	Human	GGAATCTAGAACATATACTAAAATTGGAAC	GGAACTCGAGTTTGCGTGTCATCCTTGCGC	
**5S rRNA**	Human	GGCCATACCACCCTGAACGC	CAGCACCCGGTATTCCCAGG	
**7SL**	Human	CAAAACTCCCGTGCTGATCA	GGCTGGAGTGCAGTGGCTAT	
MALAT1	Human	GTCATAACCAGCCTGGCAGT	GCTTATTCCCCAATGGAGGT	
***ECE1* mRNA**	Human	CGGAGCACGCGAGCTATGA	CCGCCAGAAGTACCACCAAC	Primers span exon-exon junction
***IPO7* mRNA**	Human	TCAATTTTGGAGGCCCAGCA	AGGTTGCATGATCAGGTCCC	Primers span exon-exon junction
MAN1A2	Human	CACATGATGATGTACAGCAGAGC	ATCGAACAGCAGGATTACCTGA	Primers span exon-exon junction
TNPO3	Human	ACATTGCAGCTCGTGTACCA	AGCATGACTCCACATCCTGC	Primers span exon-exon junction
GAPDH	Human	CAGAACATCATCCCTGCCTCTACT	GCCGAGCTTCCCGTTCA	Used to detect human genome copies
RPS13	Human	TCGGCTTTACCCTATCGACGCAG	ACGTACTTGTGCAACACCATGTGA	
**CpG DNA**	N/A	T*C*G*T*C*G*T*T*T*T*G*T*C*G*T*T*T*T*G*T*C*G*T*T (RNase Free HPLC Purification, * Phosphorothioate Bond)		Used for CpG immunostimulation

Supplementary Table 3. RNA-Seq data analyzed in this study

Overview of bulk RNA-Seq data analyzed in this study. If data from any of the sample sets was previously published we have included PubMed reference numbers.

Dataset	Model	Experiment information	Replicates	Sequencing type	SRA or GEO Accession
**HSV-1 infection**	**Lytic infection**	MRC-5 cells infected with HSV-1 strain KOS (MOI 10 PFU/cell) for 12 or 24 hours. **PMID: 36724259**	2-4	Illumina 150 PE	SRR19779319, SRR19779318, SRR19787559
	**Latent infection**	Female 8-week-old BALB/cAnTAC mice infected with HSV-1 strain 17 (10^5 PFU) via the ocular route. Latently infected trigeminal ganglion (TG) were harvested 4 weeks after primary infection.	4	Illumina 150 PE	SRR19792335, SRR19792334
	**Lytic reactivation**	Female 8-week-old BALB/cAnTAC mice infected with HSV-1 strain 17 (10^5 PFU) via the ocular route. Trigeminal ganglia were explanted (TG explant) ~4 weeks after primary infection in the presence of vehicle or 2 uM JQ1 for 12 hours.	2-3	Illumina 150 PE	SRR25824398, SRR25824397, SRR25824394, SRR25824396
**HCMV infection**	**Lytic infection**	MRC-5 cells infected with HCMV strain TB40/E (MOI 3 PFU/cell) for 24 or 72 hours. **PMID: 28874566**	2	Illumina 100 PE	SRR5629593, SRR5629594, SRR5629591, SRR5629592, SRR5629589, SRR5629590, SRR5629587, SRR5629588, SRR5629577, SRR5629578, SRR5629575, SRR5629576, SRR5629573, SRR5629574, SRR5629571, SRR5629572
**KSHV infection**	**Lytic infection**	LEC infected with KSHV strain BAC16 (MOI 1 PFU/cell) for 24 or 72 hours. **PMID: 36724259**	2	Illumina 150 PE	SRR20020769, SRR25816558, SRR20020770
	**Lytic reactivation**	iSLK-BAC16 induced with 1 ug/mL Doxycycline 1 mM Sodium Butyrate for 24 or 72 hours. **PMID: 36724259**	4	Illumina 150 PE	SRR25816557, SRR20020761, SRR25816556, SRR20020757, SRR20020758
**MHV68 infection**	**Lytic infection**	NIH 3T3 cells infected with MHV68 strain H2B-YFP (MOI 5 PFU/cell) for 6 or 18 hours.	2	Illumina 150 PE	SRR19792326, SRR25823339, SRR19792325
	**Lytic reactivation**	A20 HE-RIT cells induced with 5 ug/ml Dox and 20 ng/ml TPA for 6 or 24 hours.	2	Illumina 150 PE	SRR19792324, SRR25823338, SRR19792321
**Interferon stimulation**	**Uninfected**	MRC-5, LEC, or Akata- cells treated with IFN-β or -γ or- for 48 hours.	3	Illumina 150 PE	SRR25905055, SRR25905049, SRR25905048, SRR25905050, SRR25905054, SRR25905051, SRR25905053, SRR25905052
**EBV infection**	**Latent infection**	Akata+ (EBV positive) or Akata- (EBV negative) cells. 074301 Arraystar Human CircRNA microarray V2. **PMID: 36724259**	3	Microarray	GSE206824

SUPPLEMENTARY FIGURES

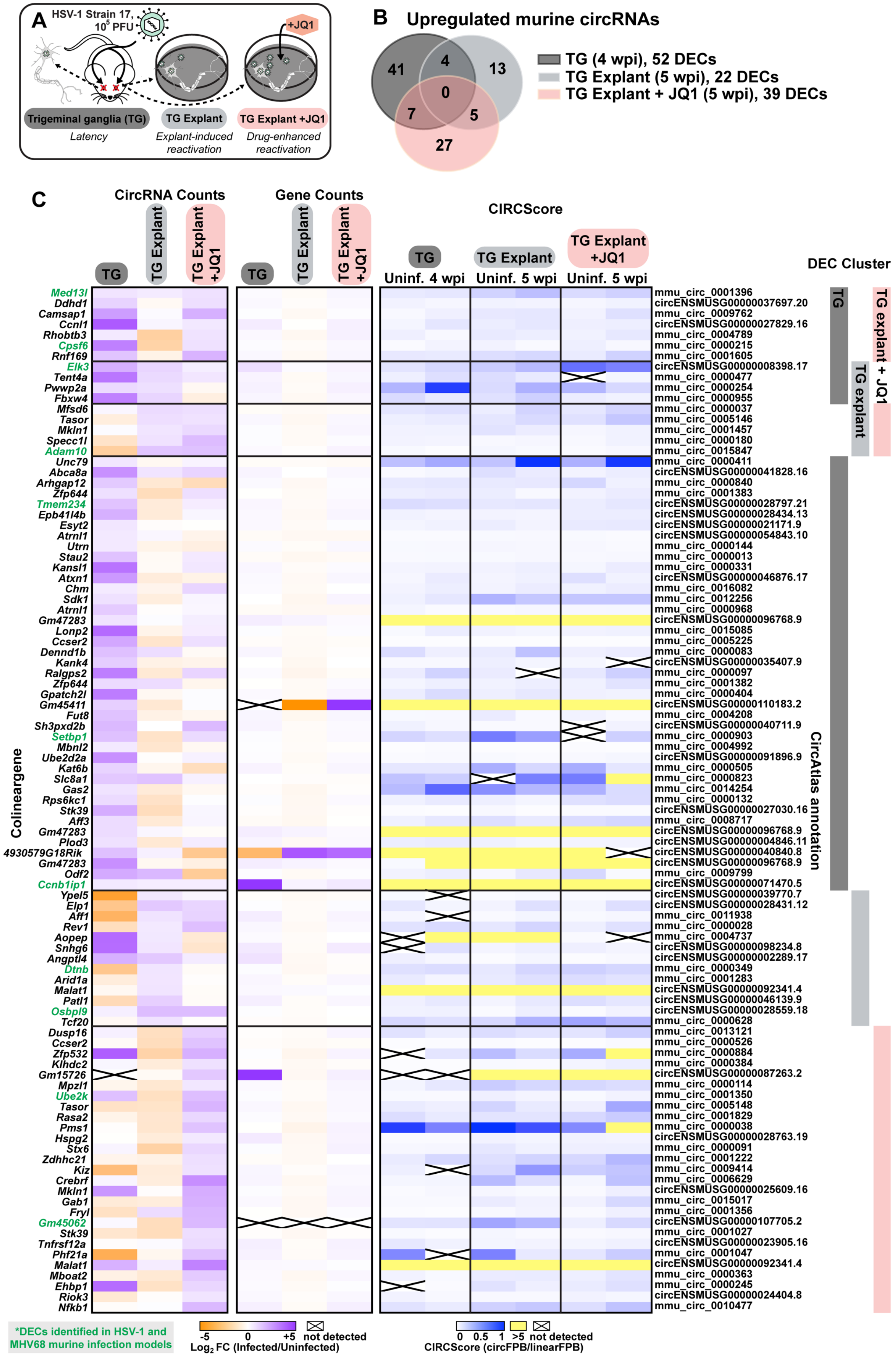

Supplementary Figure 1-1. Murine circRNAs upregulated in HSV-1 infection models.
A) Infographic for murine HSV-1 infection models used in this study. B) Overlap of upregulated murine circRNA detected by bulk RNA-Seq from HSV-1 latency (TG, n=4), explant-induced reactivation (TG explant, n=3), and drug-enhanced reactivation (TG explant + JQ1, n=2). Upregulated circRNAs had a raw BSJ count across the sample set >10, $Log_2FC>0.5$, and RankProduct p-value <0.05. C) Heatmaps for upregulated murine circRNAs, plotted as circRNA counts (Log_2FC Infected/Uninfected ERCC normalized BSJ counts), Gene counts (Log_2FC Infected/Uninfected ERCC normalized gene counts), or CIRCScore (circFPB/linearFPB). Heatmap values are the average of biological replicates. Log_2FC is relative a paired uninfected control. Differentially expressed circRNAs (DECs) from murine HSV-1 and MHV68 infection models have their colinear gene names colored green (11 overlapping DECs).

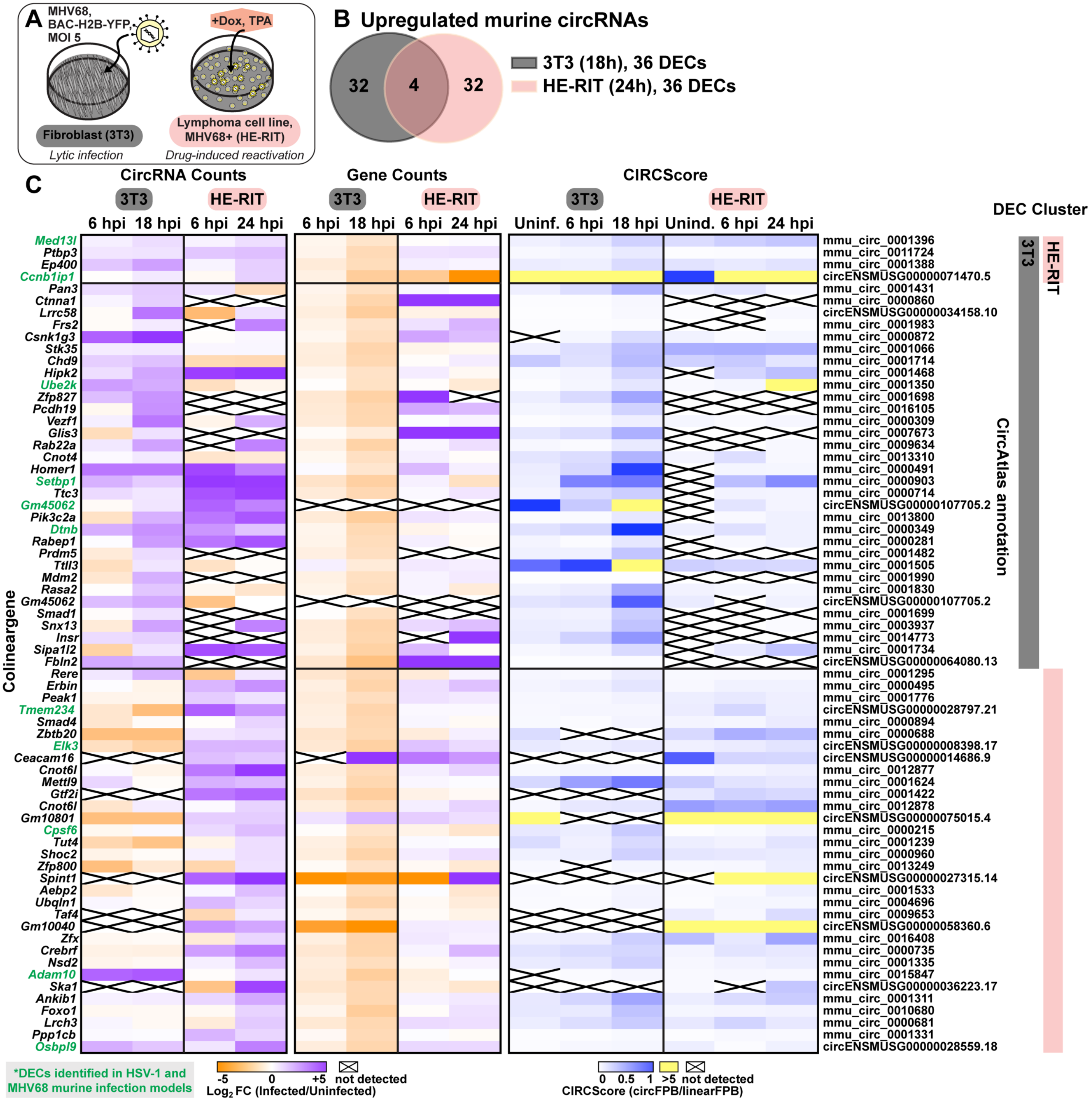

Supplementary Figure 1-2. Murine circRNAs upregulated in MHV68 infection models.
A) Infographic for murine MHV68 infection models used in this study. B) Overlap of upregulated murine circRNA detected by bulk RNA-Seq from MHV68 *de novo* infection (NIH 3T3, n=2) and drug-induced reactivation (HE-RIT, n=2). Upregulated circRNAs had a raw BSJ count across the sample set >10, $Log_2FC>0.5$, and p-value <0.05. C) Heatmaps for upregulated murine circRNAs, plotted as CircRNA counts (3T3: Log_2FC Infected/Uninfected ERCC normalized BSJ counts, HE-RIT: Log_2FC Drug treated/Uninduced ERCC normalized BSJ counts), Gene counts (3T3: Log_2FC Infected/Uninfected ERCC normalized BSJ counts, HE-RIT: Log_2FC Drug treated/Uninduced ERCC normalized BSJ counts), or CIRCScore (circFPB/linearFPB). Heatmap values are the average of biological replicates. Log_2FC is relative a paired uninfected or uninduced control. Differentially expressed circRNAs (DECs) from murine HSV-1 and MHV68 infection models have their colinear gene names colored green (11 overlapping DECs).

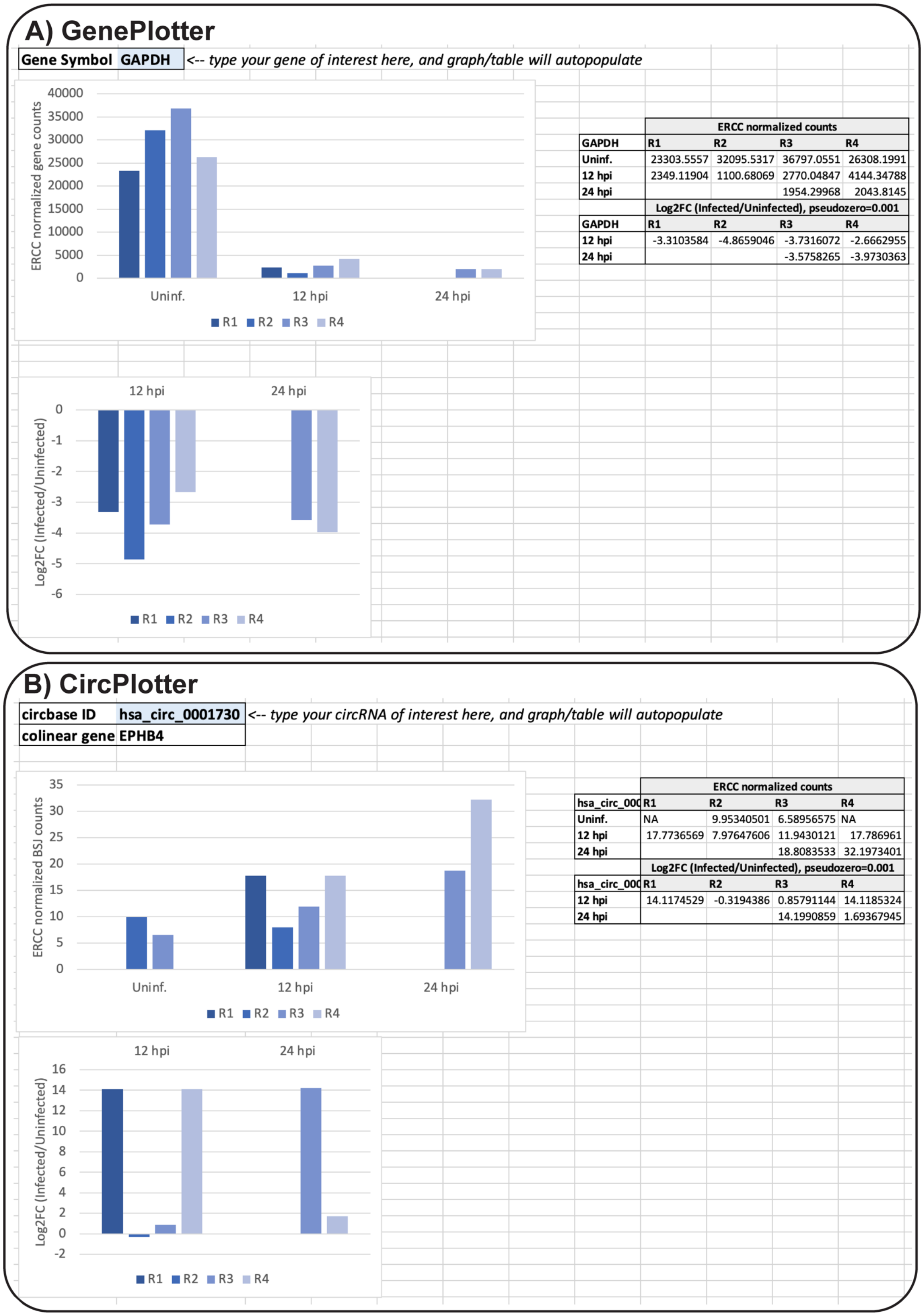

Supplementary Figure 1-3. Screenshot of interactive resource table for transcriptomic data. Supporting datasets were generated for all bulk RNA-Seq data analyzed in this study. Datasets include raw and normalized gene and BSJ counts. Users can query any gene or circRNA of interest using the GenePlotter of CircPlotter tabs.

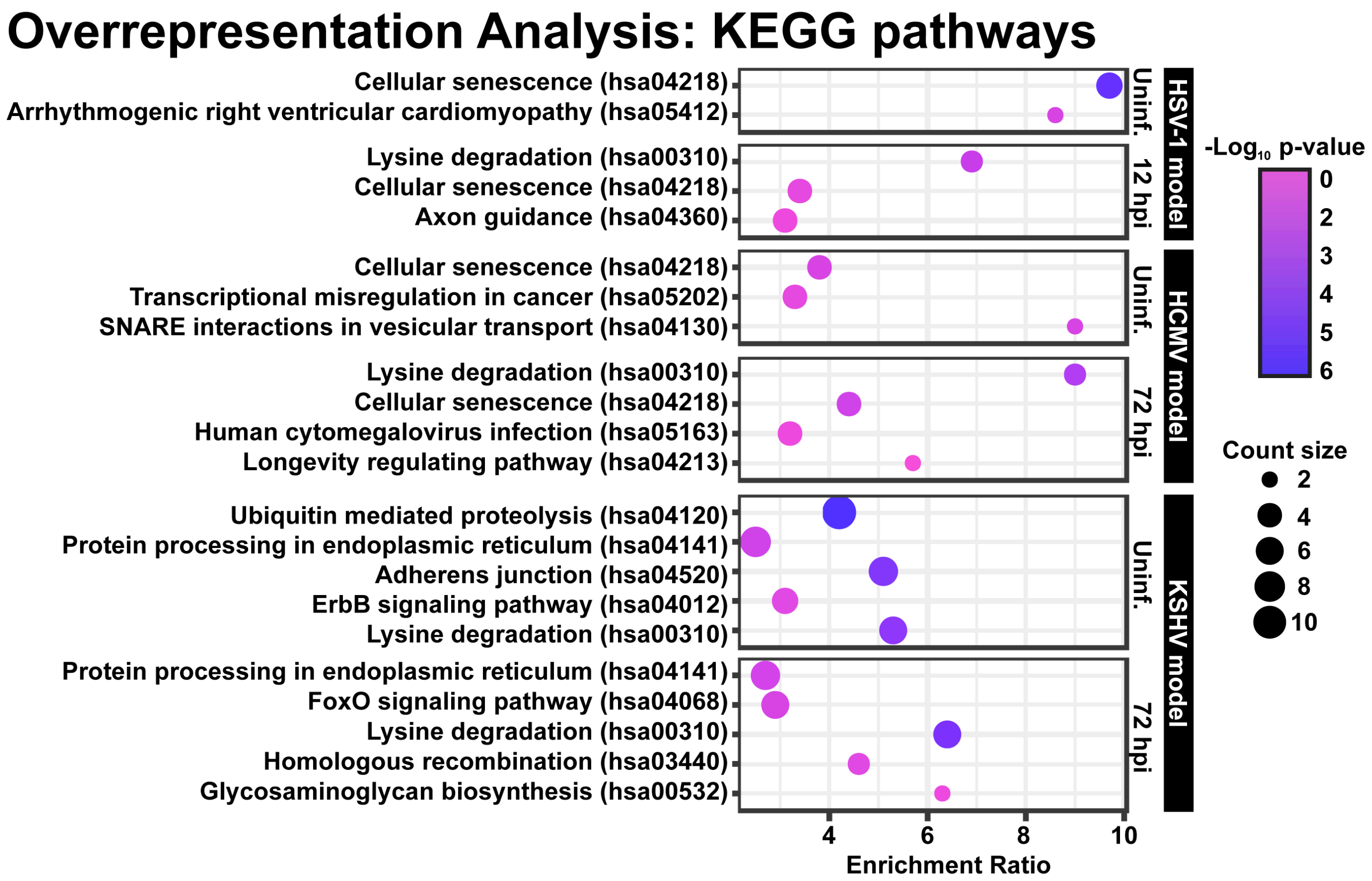

Supplementary Figure 1-4. Overrepresentation analysis (ORA) of colinear genes containing circRNAs expressed in human herpesvirus infection models. ORA was performed using a list of genes colinear to circRNAs detected in a given model (>10 raw BSJ read counts). Data is represented as a pathway enrichment bubble plot, with bubble size indicating the number of genes from a given pathway, y-axis is enrichment ratio (relative to expected from a random gene sampling), and bubble color is $-\text{Log}_{10}$ p-value. Only pathways with a p-value of <0.05 were included.

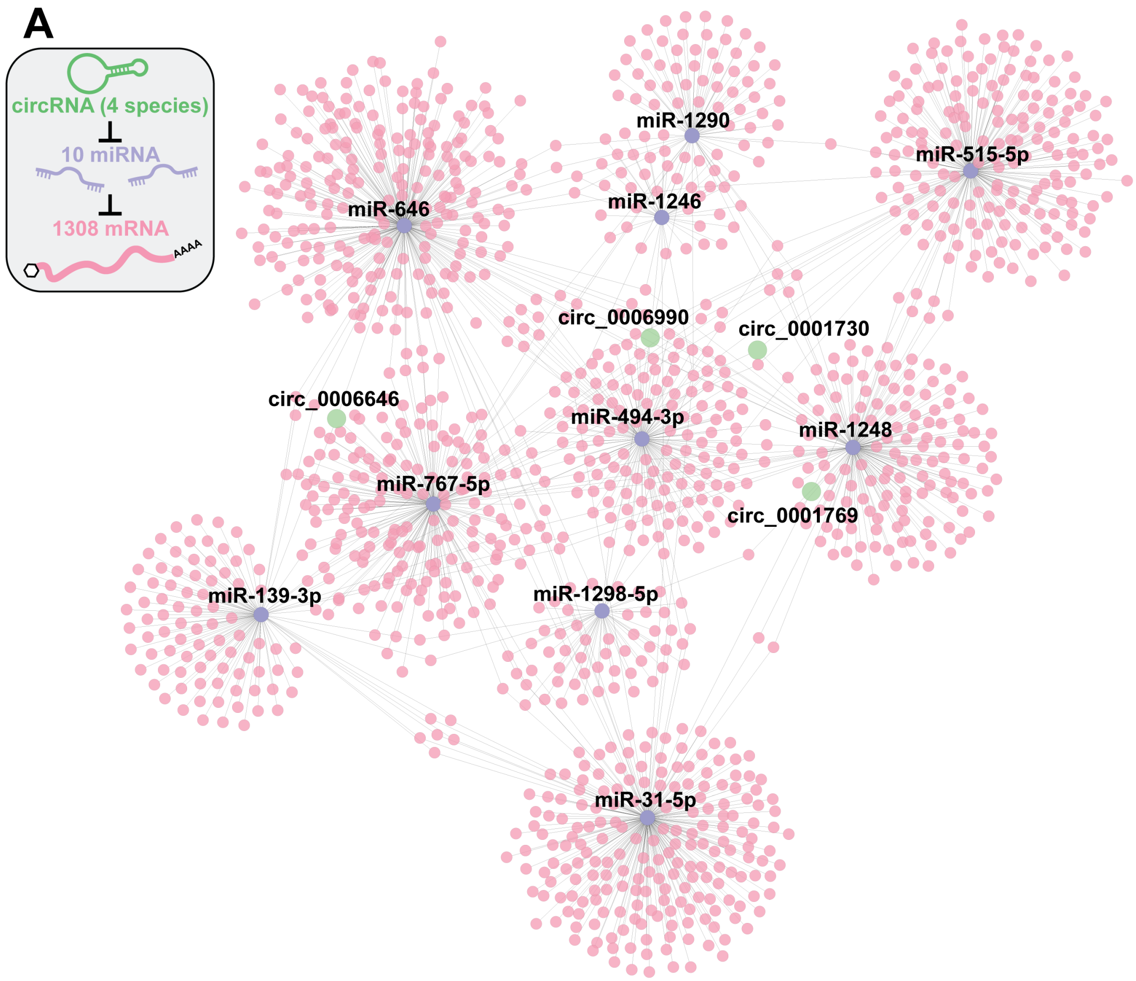

B miEAA-miRNA Enrichment Annotation

MHC class I protein complex assembly (GO0002397), p-value=2.06e-5
hsa-miR-1248; hsa-miR-515-5p; hsa-miR-1246; hsa-miR-1290; hsa-miR-1298-5p; hsa-miR-646; hsa-miR-31-5p

MHC class Ib protein complex assembly (GO0002398), p-value=2.06e-5
hsa-miR-1248; hsa-miR-515-5p; hsa-miR-1246; hsa-miR-1290; hsa-miR-1298-5p; hsa-miR-646; hsa-miR-31-5p

TAP2 binding (GO0046979), p-value=3.33e-5
hsa-miR-1248; hsa-miR-515-5p; hsa-miR-1246; hsa-miR-1290; hsa-miR-1298-5p; hsa-miR-646; hsa-miR-31-5p

TAP complex binding (GO0062061), p-value=3.35e-5
hsa-miR-1248; hsa-miR-515-5p; hsa-miR-767-5p; hsa-miR-1246; hsa-miR-1290; hsa-miR-1298-5p; hsa-miR-139-3p; hsa-miR-646; hsa-miR-31-5p

Tapasin-ERp57 complex (GO0061779), p-value=2.57e-5
hsa-miR-1248; hsa-miR-515-5p; hsa-miR-1246; hsa-miR-1290; hsa-miR-1298-5p; hsa-miR-494-3p; hsa-miR-646; hsa-miR-31-5p

Peptide antigen stabilization (GO0050823), p-value=2.06e-5
hsa-miR-1248; hsa-miR-515-5p; hsa-miR-1246; hsa-miR-1290; hsa-miR-1298-5p; hsa-miR-646; hsa-miR-31-5p

Supplementary Figure 1-5. In silico network predictions for circRNA-miRNA-mRNA networks. A) Four commonly-regulated human circRNAs (hsa_circ_0001730, hsa_circ_0006990, hsa_circ_0006646, hsa_circ_0001769) were examined for miRNA-binding sites using CircInteractome (1). Experimentally supported miRNA-mRNA interactions were then added to the network based on DIANA-TarBase v8.0 (2). Cytoscape 3.10 was used for visualizing the network. B) Overrepresentation analysis of predicted target miRNAs was performed with miEAA2.0 (4). Enriched Gene Ontology terms (http://geneontology.org), p-values, and miRNAs that were predicted to be regulated by circRNAs are shown.

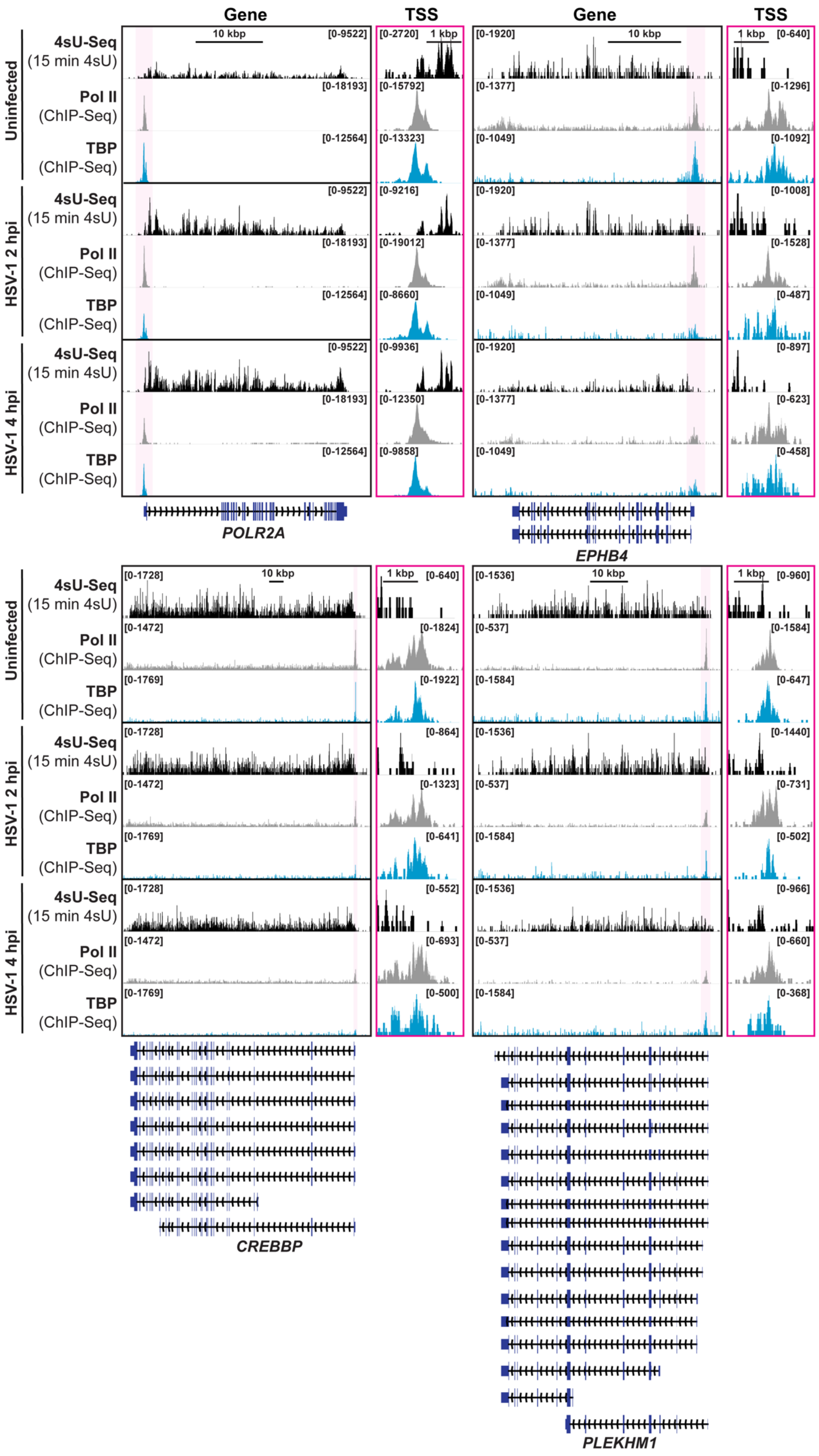
Gene
TSS
Gene
TSS
Uninfected
HSV-1 2 hpi
HSV-1 4 hpi
4sU-Seq
(15 min 4sU)
Pol II
(ChIP-Seq)
TBP
(ChIP-Seq)
10 kbp
1 kbp
POLR2A
EPHB4
CREBBP
PLEKHM1

Supplementary Figure 3-1. Transcriptional activity of genes containing circRNAs upregulated after HSV-1 infection. Nascent transcription and binding of general transcription factors for genes that host herpesvirus infection-induced circRNAs. Our previously published data from fibroblasts infected with HSV-1 (MRC-5 + KOS MOI 10) was reanalyzed (8). 4sU-Seq data is normalized to rRNA mapped reads. ChIP-Seq data is the average of biological duplicates and normalized to sequencing depth. Y-axes maximum and minimum values are listed within brackets. Traces spanning genes have the same y-axis maximums across infection conditions. Traces spanning 2.5 kbp transcription start sites (TSS) have autoscaled y-axis maximums.

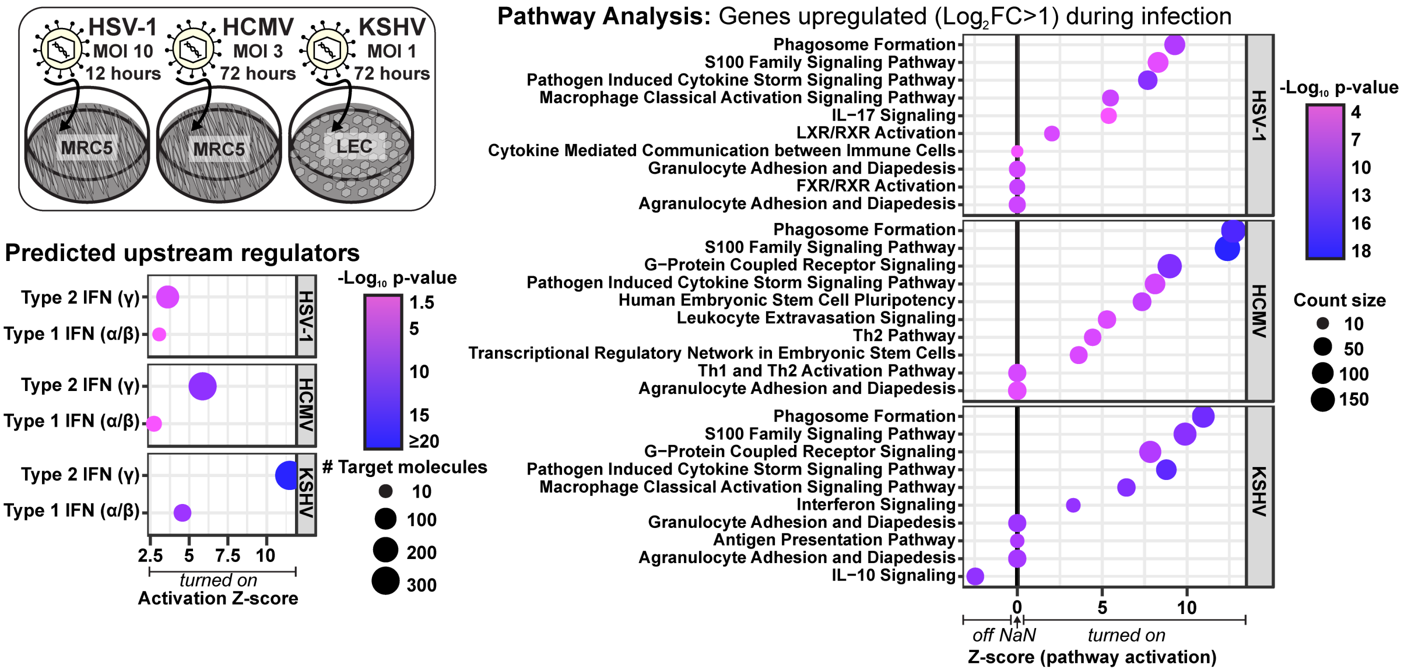

Supplementary Figure 4-1. Ingenuity pathway analysis (IPA) for upregulated genes during herpesvirus lytic infection. IPA (Qiagen) was performed on bulk RNA-Seq datasets for HSV-1 (n=4), HCMV (n=2), and KSHV (n=2). RNA-Seq data was normalized using ERCC spike-in controls. Only genes with an average $Log_2FC > 1$ (Infected/Uninfected) were used for IPA. The predicted upstream role of Type I and II interferon based on upregulated gene identities was presented in a bubble plot. Z-score indicates activation status, -log_{10}p-value indicates enrichment significance, and count size is the number of target molecules downstream of IFN that were upregulated. The top 10 most significantly (by p-value) altered pathways were plotted. Z-score indicates predicted pathway activation, -log_{10}p-value indicates enrichment significance, and count size is the number of upregulated genes in the pathway.

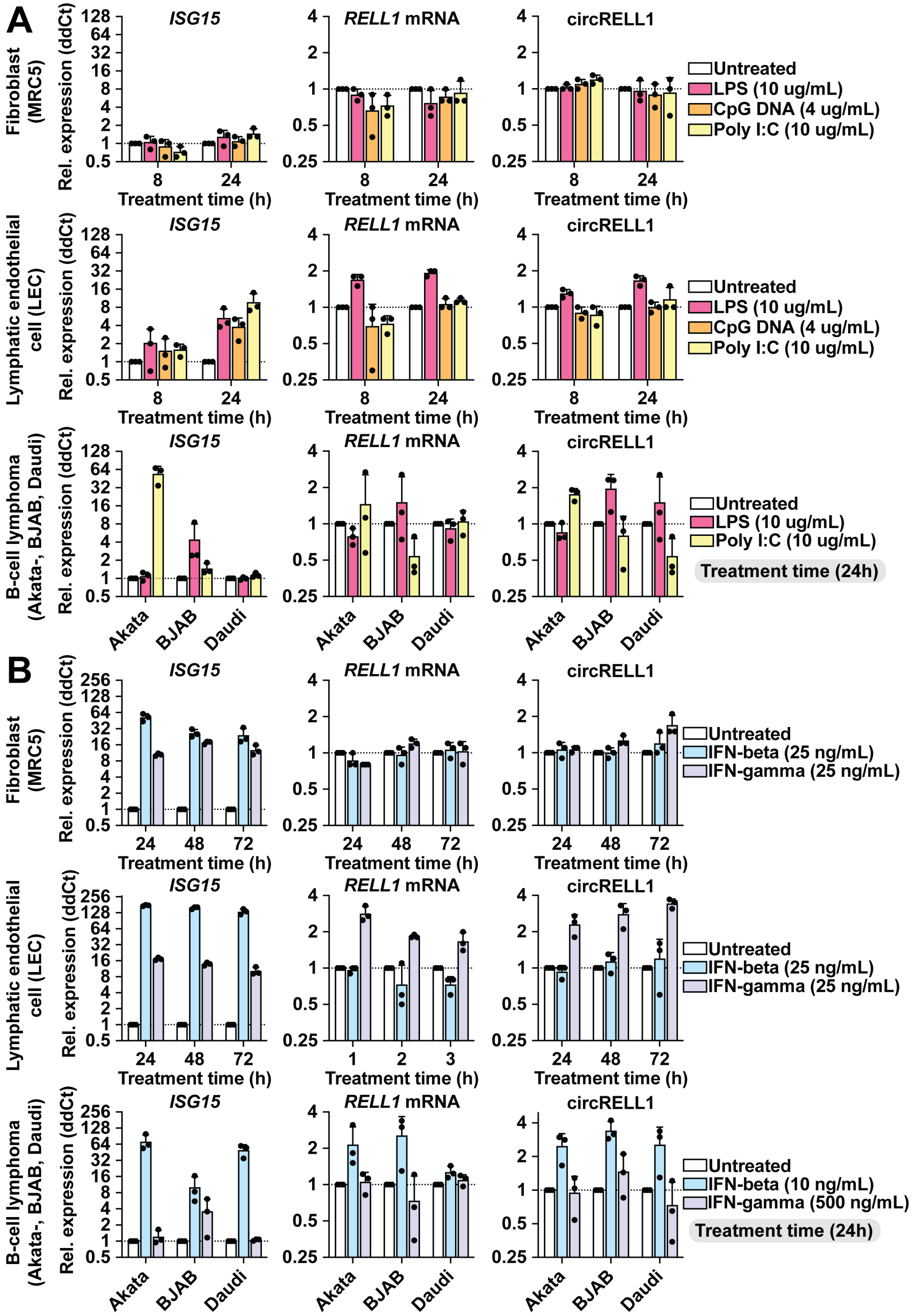

Supplementary Figure 4-2. CircRELL1 expression changes in response to immune stimuli. Fibroblasts (MRC-5), lymphatic endothelial cells (LEC), or B-cell lymphoma cells (Akata-, BJAB, Daudi) were treated with immune stimulants including A) lipopolysaccharide (LPS), poly I:C, CpG DNA, or B) IFN-β and -γ. RNA was collected and assessed after reverse transcription using qPCR. Data is plotted as relative expression (ddCt) using 18S rRNA as the reference gene, and relative to a paired untreated sample. Data points are biological replicates, column bars are the average, and error bars are standard deviation.

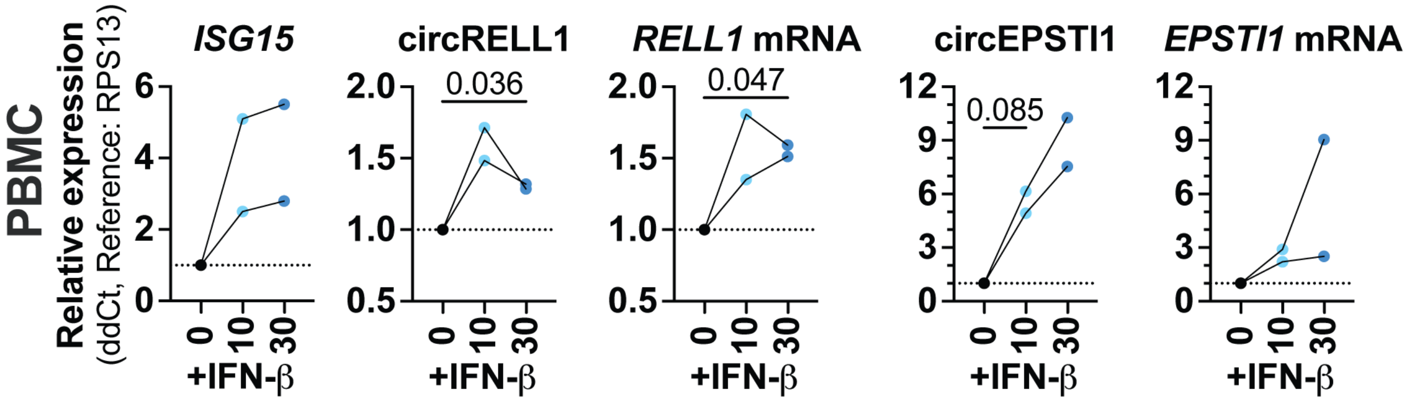

Supplementary Figure 4-3. Interferon stimulated circRNAs in PBMCs.
Human peripheral blood peripheral blood mononuclear cells (PBMCs) were isolated from two donors and treated with IFN-β (0, 10, or 30 μg/ml) for 24 hours. RNA was collected and assessed after reverse transcription using qPCR. Data is plotted as relative expression (ddCt) using *RPS13* mRNA as the reference gene, and relative to a paired untreated sample. Data points from same donors are connected by lines. Significances were calculated using paired t-tests and any p-values < 0.1 are shown.

REFERENCES

1. D. B. Dudekula *et al.*, CircInteractome: A web tool for exploring circular RNAs and their interacting proteins and microRNAs. *RNA Biol* **13**, 34-42 (2016).
2. D. Karagkouni *et al.*, DIANA-TarBase v8: a decade-long collection of experimentally supported miRNA-gene interactions. *Nucleic Acids Res* **46**, D239-d245 (2018).
3. P. Shannon *et al.*, Cytoscape: a software environment for integrated models of biomolecular interaction networks. *Genome Res* **13**, 2498-2504 (2003).
4. F. Kern *et al.*, miEAA 2.0: integrating multi-species microRNA enrichment analysis and workflow management systems. *Nucleic Acids Res* **48**, W521-w528 (2020).
5. Y. Liao, J. Wang, E. J. Jaehnig, Z. Shi, B. Zhang, WebGestalt 2019: gene set analysis toolkit with revamped UIs and APIs. *Nucleic Acids Research* **47**, W199-W205 (2019).
6. X. K. Ma *et al.*, CIRCexplorer3: A CLEAR Pipeline for Direct Comparison of Circular and Linear RNA Expression. *Genomics Proteomics Bioinformatics* **17**, 511-521 (2019).
7. A. Oberstein, T. Shenk, Cellular responses to human cytomegalovirus infection: Induction of a mesenchymal-to-epithelial transition (MET) phenotype. *Proc Natl Acad Sci U S A* **114**, E8244-e8253 (2017).
8. S. E. Dremel, F. L. Sivrich, J. M. Tucker, B. A. Glaunsinger, N. A. DeLuca, Manipulation of RNA polymerase III by Herpes Simplex Virus-1. *Nature Communications* **13**, 623 (2022).
